# Erroneous detection of desensitization doses in the prevention of hypersensitivity reactions

**DOI:** 10.1186/s13104-023-06278-2

**Published:** 2023-02-03

**Authors:** Razvan Costin Stan

**Affiliations:** Department of Biomedical Sciences, Chonnam National University, Seoyang-ro 264, Hwasun, 58128 South Korea

**Keywords:** Drug desensitization, Signal processing, IgE, Aliasing, Allergens

## Abstract

**Background:**

Desensitization protocols have empirically established their efficacy and safety in eliminating most of the hypersensitivity reactions to drugs and other allergens. Without such procedures, the offending drugs can otherwise be lethal, for some patients, when singularly administered at therapeutic doses. These binding events and the subsequent signaling cascades have been extensively modulated by different desensitization methods, without any clear explanation as to why it is necessary to use increasing allergen doses.

**Purpose:**

To use a novel theoretical approach in order to model the desensitization algorithms currently in practice, that seeks to shed light on the mechanism behind their clinical efficacy.

**Method:**

An approach using signal processing concepts is applied in this work to introduce aliasing as the erroneous detection of higher drug doses responsible for the efficacy of desensitization procedures.

**Results:**

Available experimental data is modeled and correct predictions as to the efficacy of the drug treatment procedures are produced.

**Conclusions:**

Desensitization algorithms may benefit from using concepts from signal processing theory in order to avoid hypersensitivity reactions.

## Introduction

Mast cells are responsible for immunoglobulin E (IgE)-associated hypersensitivity reactions to allergens and for other allergic disorders [1]. IgE binds with a 1:1 stoichiometry to its Fc epsilon RI (FcεRI) receptor on the surface of mast cells at a very high affinity (dissociation constant of between 10^-9^ and 10^-10^ M), which results in immediate allergic or inflammatory response upon exposure to environmental antigens [2]. Cross-linking of two or more FcεRI-bound IgE molecules by bivalent or multivalent antigens triggers, *inter alia*, the exocytosis of allergic mediators (degranulation), regulation of gene expression for chemokines/cytokines, and mast cell migration [3]. The expression of FcεRI on the surface of mast cells, and the processes that regulate its therein presence (i.e. FcεRI stabilization by IgE attachment or its recycling back to the surface) are thus critical for these cells to mount optimal responses to antigens. Unoccupied FcεRI complexes are not stable on the cell surface and are eventually endocytosized. Furthermore, FcεRI clusters produced by low-affinity antigens are slower and larger than those induced by higher affinity antigens [1], nuancing the degree of downstream signaling to the concentrations of antigens. Additionally, recycling of unoccupied FcεRI to the cell surface increases the probability of capturing circulating IgE molecules. It is therefore essential for an adaptive immune response to allergens that there be an appropriate amount of IgE-FcεRI complexes present for physiological and not pathological signaling [4]. Mast cells with higher FcεRI expression levels on their surface will mount stronger responses to antigens. It is uncertain, however, whether this is relation is linear, as both amplificatory and inhibitory loops of allergen signaling can be encountered in the functioning of mast cells [5].

The challenge that has not been addressed in the studies on the allergen detection system, therefore, is how the latter gauges antigen stochastic changes in concentration over time. It is evident that an appropriate filter has to be in place such that excessive stimulation will elicit a proper immune response, while random noise will not. In particular, it is unknown why desensitization procedures (DP) used against offending antigens are effective in preventing allergic reactions. In these non-physiological situations, allergens are delivered at regular intervals, in fixed amounts, and in strictly increasing doses. In the clinical practice, DP induce a refractive, hypo-sensitive but temporary response state to an offending drug. This is beneficial for most patients, when the allergenic drug is essential to therapy and its avoidance will lead to impaired drug management or reduced life expectancy [6]. For certain patients, however, the DP may still elicit an allergenic response and pose a health hazard at particular steps during these procedures. Despite the important medical implications, only empirical protocols are in place to reduce the effect of full dose administration of the offending drug, by using single or multiple sub-optimal doses, with the aim to diminish its allergenic potential. DP thus rely on the establishment of short-term memory only to particular rates and dose/concentrations of the drug administration protocols [7], that still have the potential to cause adverse hypersensitive reactions.

## Methods

We here use concepts from signal processing theory [8], where the input frequency (f_i_ is defined as the dose (number of impinging antigens/unit of time) used in DP. Further, sampling frequency (f_s_) is defined as the minimum number of samples/unit of time that needs to be collected to faithfully convey a signal. It is defined by the inequality (1): $\frac{2fh}{n}\le fs\le \frac{2fl}{n-1}$ , where f_h_ and f_l_ represent the high and low frequency, respectively, between any two consecutive DP steps and “*n*” is an integer that satisfies the inequality (2): $1\le n\le fh/(fh-fl)$. By definition, the Nyquist frequency (f_N_) equals f_s_/2 and is the detection frequency that must be used to accurately represent any f_i_ without aliasing. Aliasing represents an erroneous detection as low frequencies of a signal with frequencies higher than f_N_. The aliasing frequency (f_a_) equals the absolute value of the difference between f_s_ (multiplied by the closest integer multiple *m* of f_s_) and the f_i_.

## Results and discussion

The allergen detection system must balance using an appropriate f_s_ based on initial stimulation against subsequent changes in dosage at frequencies above f_N_. The minimal requirement to establish drug tolerance involves only administering an initial single sub-optimal dose, usually many orders of magnitude lower than the final therapeutic dose. It is surmised that: 1. the initial DP dose imposes an initial f_N_; 2. f_N_ is maintained until next subsequent DP dose is administered, inducing aliasing and hypo-responsiveness; 3. Hypersensitivity reactions (HSR) occur when f_a_ > f_N_, such that accurate detection is enforced. Extensive research has shown that any signal can be properly sampled and reconstructed only if it does not contain frequency components above the f_N_ [9]. We illustrate these concepts with 3 examples of DP for the chemotherapeutic agents carboplatin [10] and mAb infliximab [11], and for the antibiotic vancomycin [12], with the protocols outlined in Tables 1, 2 and 3. Input frequency represents the cumulative doses per minute used in the study. We have not here considered the case of undersampling, when n > 1.

**Table 1:** Dosing protocol for DP using carboplatin. Integer n from inequality (2) is 1. Interval between doses is 15 min

DP step	f_i_ (μg/minute)	f_a_ (μg/minute)	f_N_ (μg/minute)	f_s_ (μg/minute)
1	1.6	–	1.65	3.3
2	4.2	0.9	1.65	3.3
3	8.3	**1.7**	**1.65**	3.3
4	16.6	0.1	1.65	3.3
5	41.6	0.3	1.65	3.3
6	83.3	**2.5**	**1.65**	3.3
7	166.6	**1.7**	**1.65**	3.3
8	333.3	**6.6**	**1.65**	3.3
9	833.3	0.8	1.65	3.3
10	1666.6	0.1	1.65	3.3
11	3333.3	0.3	1.65	3.3
12	26800	0.7	1.65	3.3

In bold, DP steps where f_a_ > f_N_ and where HSR may occur

**Table 2:** Dosing protocol for DP using infliximab. Integer n from inequality (2) is 1. Interval between doses is 15 min

DP step	f_i_ (mg/minute)	f_a_ (mg/minute)	f_N_(mg/minute)	f_s_ (mg/minute)
1	0.016	–	0.0165	0.033
2	0.06	0.0001	0.0165	0.033
3	0.2	0.002	0.0165	0.033
4	0.7	0.007	0.0165	0.033
5	2.4	**0.02**	0.0165	0.033
6	7.4	0.008	0.0165	0.033
7	24	**0.06**	0.0165	0.033
8	74	0.01	0.0165	0.033
9	240	**0.6**	0.0165	0.033
10	740	0.008	0.0165	0.033
11	2406	**0.6**	0.0165	0.033
12	7406	**0.6**	0.0165	0.033
13	16733	**0.3**	0.0165	0.033

In bold, DP steps where f_a_ > f_N_ and where HSR may occur

**Table 3:** Dosing protocol for DP using vamcomycin. Integer n from inequality (2) is 1. Interval between doses is 10 min

DP step	f_i_ (μg/minute)	f_a_ (μg/minute)	f_N_ (μg/minute)	f_s_ (μg/minute)
1	0.1	–	0.1	0.2
2	0.43	0.03	0.1	0.2
3	1.43	0.01	0.1	0.2
4	4.7	0.1	0.1	0.2
5	14.7	0.1	0.1	0.2
6	48	0	0.1	0.2
7	148	0	0.1	0.2
8	478	0	0.1	0.2
9	1480	0	0.1	0.2
10	3700	0	0.1	0.2

For DP procedures against mAb, the most commonly used protocol has 12 steps but is associated with an increased risk of HSR during the very last dose administration [13]; this phenomenon is also present in our simulations (Table 2).

It has been suggested that the modulation of the time interval between doses may be more relevant for successful DP procedures, rather than increasing the dosage [14]. Based on the data from Table 3 [11], an increase in administration time to 15 min between doses has been introduced, and the parameters of interest recalculated, as shown in Table 4. In so doing, the possibility of HSR is reintroduced at specific DP steps.

**Table 4:** Dosing protocol for DP using vamcomycin. Integer n from inequality (2) is 1. Interval between doses has been modified to 15 min

DP step	f_i_ (μg/minute)	f_a_ (μg/minute)	f_N_ (μg/minute)	f_s_ (μg/minute)
1	0.06	–	0.06	0.12
2	0.28	0.04	0.06	0.12
3	0.95	0.1	**0.06**	0.12
4	3	0	0.06	0.12
5	9.8	0.1	**0.06**	0.12
6	32	0.8	**0.06**	0.12
7	98	0.1	**0.06**	0.12
8	318	0	0.06	0.12
9	986	0.8	**0.06**	0.12
10	2466	0	0.06	0.12

In bold, DP steps where f_a_ > f_N_ and where HSR may occur

The appearance of HSR also appears to be skewed depending on the class of drugs, with monoclonal antibodies being more immunogenic [15]. Additionally, the initial setting of the f_s_ may suppress the establishment of f_a_. In the above examples, it is notable that the first dose does not have an alias frequency, as the sampling frequency is set post-stimulation.

## Conclusions

Aliasing is an otherwise undesirable effect that causes different higher frequency signals to become falsely sampled as having lower frequencies. We propose that this mechanism may be contribute to the clinical efficacy of DP, where the artificial nature of the procedures ensures that the f_s_ of the previous DP step (other than the first dose) is used to sample the subsequent dose. Alterations in alias frequency that exceed the values of Nyquist frequency may lead to HSR observed. The challenge remains to establish proper DP that modulate aliasing frequencies for different class of drugs, such that HSR be prevented.

## Limitations

- the case of undersampling has not been considered in this study.
- due to clinical concerns, there are no datasets available whereby the intervals between doses had been systematically varied for the same patients
- there are studies where the initial dose was varied within a wide range
- the short-term memory that underscores the continuous use of f_s_ from a particular dose to the subsequent dose is hypothesized, although IgE- FcεRI complexes themselves can themselves have a long half-life of approx. 8 days ex vivo [16].

## Data Availability

The datasets analyzed during the current study are available in references 11–13.

## Abbreviations

IgE: Immunoglobulin E
FcεRI: Fc epsilon receptor I
DP: Desensitization procedures
f_i_: Input frequency
f_s_: Sampling frequency
f_N_: Nyquist frequency
HSR: Hypersensitivity reactions
